# Local Ancestry to Identify Selection in Response to Trypanosome Infection in Baoulé x Zebu Crossbred Cattle in Burkina Faso

**DOI:** 10.3389/fgene.2021.670390

**Published:** 2021-09-27

**Authors:** Bernadette Yougbaré, Dominique Ouédraogo, Arnaud S. R. Tapsoba, Albert Soudré, Bienvenue L. Zoma, Pablo Orozco-terWengel, Sanou Moumouni, Salifou Ouédraogo-Koné, Maria Wurzinger, Hamidou H. Tamboura, Amadou Traoré, Okeyo Ally Mwai, Johann Sölkner, Negar Khayatzadeh, Gábor Mészáros, Pamela A. Burger

**Affiliations:** ^1^Department of Sustainable Agricultural Systems, University of Natural Resources and Life Sciences Vienna (BOKU), Vienna, Austria; ^2^Institut de l’Environnement et de Recherches Agricoles (INERA), Ouagadougou, Burkina Faso; ^3^Institut du Développement Rural, Université Nazi Boni, Bobo-Dioulasso, Burkina Faso; ^4^Unité de Formation et de Recherche en Sciences et Technologies, Université Norbert Zongo, Koudougou, Burkina Faso; ^5^School of Biosciences, Cardiff University, Cardiff, United Kingdom; ^6^International Livestock Research Institute (ILRI), Nairobi, Kenya; ^7^SUISAG, Sempach, Switzerland; ^8^Research Institute of Wildlife Ecology, Vetmeduni Vienna, Savoyenstraße 1, Vienna, Austria

**Keywords:** admixture, local ancestry deviation, selection signature, SNP, *F*st, cattle, Burkina Faso

## Abstract

The genomes of crossbred (admixed) individuals are a mosaic of ancestral haplotypes formed by recombination in each generation. The proportion of these ancestral haplotypes in certain genomic regions can be responsible for either susceptibility or tolerance against pathogens, and for performances in production traits. Using a medium-density genomic marker panel from the Illumina Bovine SNP50 BeadChip, we estimated individual admixture proportions for Baoulé x Zebu crossbred cattle in Burkina Faso, which were tested for trypanosome infection by direct ELISA from blood samples. Furthermore, we calculated local ancestry deviation from average for each SNP across 29 autosomes to identify potential regions under selection in the trypanotolerant Baoulé cattle and their crossbreds. We identified significant deviation from the local average ancestry (above 5 and 10% genome-wide thresholds) on chromosomes 8 and 19 in the positive animals, while the negative ones showed higher deviation on chromosomes 6, 19, 21, and 22. Some candidate genes on chromosome 6 (*PDGFRA*) and chromosome 19 (*CDC6*) have been found associated to trypanotolerance in West African taurines. Screening for *F*_*ST*_ outliers in trypanosome positive/negative animals we detected seven variants putatively under selection. Finally, we identified a minimum set of highly ancestry informative markers for routine admixture testing. The results of this study contribute to a better understanding of the genetic basis of trypanotolerance in Baoulé cattle and their crossbreeds. Furthermore, we provide a small informative marker set to monitor admixture in this valuable indigenous breed. As such, our results are important for conserving the genetic uniqueness and trypanotolerance of Baoulé cattle, as well as for the improvement of Baoulé and Zebu crossbreds in specific community-based breeding programs.

## Introduction

The *Bos taurus taurus* and *Bos taurus indicus* subspecies are the two most important cattle types in West Africa (Belemsaga et al., 2005; Okeyo Mwai et al., 2015). These animals have been raised in complex social and political processes, and they have adapted to harsh environmental conditions over the centuries (Dueppen, 2012). The adaptive traits include the tolerance to diseases and drought, ability to walk long distances, and capacity to survive on poor pastures (Okeyo Mwai et al., 2015). Yet, this valuable diversity is increasingly threatened by genetic dilution due to changes in production systems, livestock herders’ preferences for specific breeds and/or traits, market conditions and other opportunities (Hanotte et al., 2010). The taurine cattle, more specifically the Baoulé cattle have existed in the tsetse fly (Glossina spp.) challenged zones for long and therefore acquired trypanotolerance, an immunology phenomenon that has a genetic basis (Naessens et al., 2002; Agyemang, 2005). These animals have a capacity to rid themselves of trypanosome parasites and maintain low parasitemia. Thus, trypanotolerant animals have been introduced in other tsetse affected countries of Africa to make use of their genetic advantage in purebred populations or crossed to other types like Zebu. Several studies revealed admixture among the taurine and Zebu subspecies (Hanotte, 2002; Freeman et al., 2004; Flori et al., 2014) as the result of the continuous genetic flow that occurs every year during seasonal cross-border livestock movements from the drier Sahelian zones in the north to the more humid zones in the south of West and East Africa.

The south-western region of Burkina Faso is the original habitat of Baoulé cattle. In this area, production systems are mixed crop-livestock and agroforestry, with the Lobi ethnic group concentrating on subsistence crop production while the transhumant people tend to keep their lifestyle of pastoral livestock production. The cattle population in this region is estimated at 343,000 heads, representing about 4% of the estimated national stock of 9 million according to the Ministry of Animal Resources (Ministere des ressources animales, 2014). The livestock system is extensive in all studied departments (Zoma et al., 2020), with 7 to 100 cattle per farmers. The indigenous Baoulé cattle, despite its small size and lower growth rate, is well adapted to the local environment of West Africa. It has gained cultural importance due to its social roles and tolerance to trypanosomiasis (Zoma et al., 2020). However, the continued crossbreeding with Zebu cattle because of its large size threatens the integrity of the Baoulé breed (Yougbaré et al., 2020). Recently, community-based breeding programs have been implemented in the south-western region of Burkina Faso to conserve and improve the local genetic resources of the indigenous Baoulé cattle as well as Baoulé x Zebu advanced crosses (Ouédraogo et al., 2020; Zoma et al., 2020).

Since the advent of high-throughput single nucleotide polymorphism (SNP) genotyping, inferring selection signatures from differences in local admixture levels has received considerable attention in human genetics (Tang et al., 2007; Jin et al., 2012; Bhatia et al., 2014). Similar studies in livestock investigated local ancestry levels of New World Creole cattle (Gautier and Naves, 2011; Flori et al., 2014; Pitt et al., 2018) and selection signatures in dairy cattle in East Africa, resulting from admixture of European breeds (Kim and Rothschild, 2014), as well as in East African short horn Zebu (Bahbahani et al., 2015).

The genomic ancestry proportions between trypano-susceptible indicine Zebu and the trypano-tolerant taurine Baoulé cattle in Burkina Faso were assessed based on microsatellites and 155 SNPs in 23 candidate regions (Smetko et al., 2015). In this study, we followed up and extended on the previous work using dense genomic marker data. Our study aimed to estimate the individual local ancestry proportions for each SNP to identify potential regions under selection in Baoulé x Zebu crossbreds tested positive or negative for trypanosomosis and finding a small set of ancestry informative SNP for routine admixture testing. Estimating the proportional contributions of ancestral populations in admixed (crossbred) individuals is important to clarify the population structure, historical background, and pattern of admixture along the genome of admixed individuals.

## Materials and Methods

### Study Areas and Sample Collection

**FIGURE 1:**
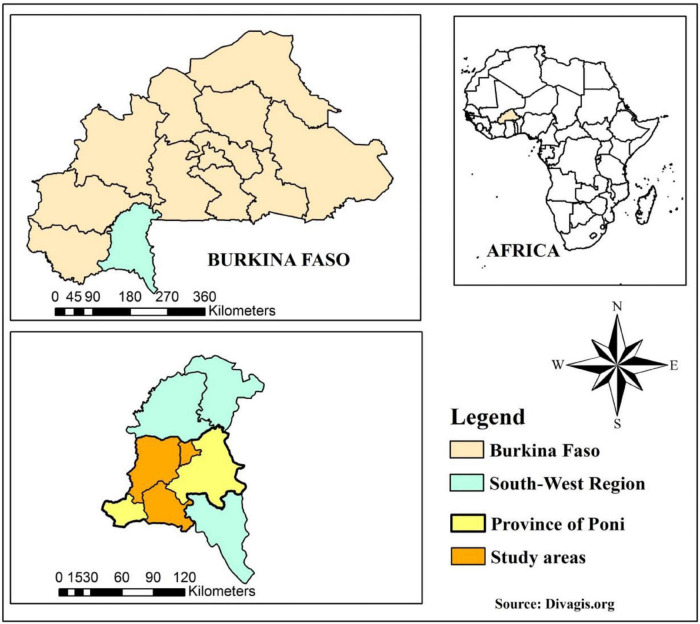
Map of Burkina Faso showing the three study areas.

This study was carried out in the province of Poni in the South-western administrative region of Burkina Faso. We selected three study sites with different management and breeding systems of Baoulé cattle and Baoulé x shorthorn Zebu crossbreds, including 27 villages from the Bouroum-Bouroum, Kampti and Loropéni departments (Figure 1). In the Bouroum-Bouroum department, we worked with 55 sedentary farmers of the ethnic group of Lobi, who keep purebred Baoulé and are the owners of these animals. In Kampti, we included 18 farms with mostly Baoulé x Zebu crossbreds and some pure Baoulé owned by the Mossi ethnic group, but herded by the transhumant Fulani people. Finally, in Loropéni 15 farms were included mainly with crossbred animals kept by Lobi and Djan breeders. As all samples were collected within close geographic distances (< 50 km) in the tsetse infested province of Poni, it is reasonable to assume that all individuals were exposed to the same trypanosome infection challenge.

A total of 737 blood samples, including 387 Baoulé and Baoulé x Zebu crossbreds from Bouroum-Bouroum, and 350 crossbreeds from Kampti and Loropéni were collected in EDTA tubes during the health monitoring activities of the “Characterization and Sustainable Utilization of Local Cattle Breeds” (LoCaBreed) project. DNA extraction from EDTA blood samples was performed with the MasterPureTM DNA Purification Kit for Blood Version II (Biozym Scientific, Oldendorf, Germany) following the manufacturer’s protocol. The trypanosomosis status was recorded by indirect ELISA test to diagnose positive or negative trypanosome infection in the blood samples (Desquesnes et al., 2003) resulting in a total of 377 positive and 360 negative animals (Table 1).

**TABLE 1:** The 737 genotyped cattle with the trypanosomosis status.

**Regions**	**Negative animals**	**Positive animals**	**Total**
Bouroum-Bouroum	204	183	387
Kampti	87	89	176
Loropéni	69	105	174
Total	360	377	737

### Genotype Data

The genotyping of the 737 DNA samples with the Illumina Bovine SNP50 BeadChip was performed at Neogen (Lincoln, United States). Additional genotypes from 30 purebred Zebu and 35 crossbreds (Pérez et al., 2014) were included in the study to represent the two ancestral populations (Baoulé and Zebu) and to increase the number of crossbreds, summing to a total of 802 animals. Stringent quality filtering of the data was performed with PLINK 1.9 (Purcell et al., 2007; Chang et al., 2015). Specifically, the dataset was controlled to exclude non-autosomal SNPs, and SNPs with a minor allele frequency (MAF) lower than 0.05, a call rate less than 90% and those that deviated from Hardy Weinberg equilibrium with Fisher’s exact test with *P*-value 1 × 10 − 6. After quality control, 28,034 SNPs and 776 animals were available for subsequent analyses.

### Global Admixture Analysis

Unsupervised global ancestry estimation was performed with the full set of quality controlled SNPs using ADMIXTURE software (Alexander et al., 2009) with the number of ancestral populations (Baoulé and Zebu) fixed at two (*K* = 2). The admixture bar plots for ancestry proportions were created in R with the *barplot* function (The R Development Core Team, 2020). We calculated the frequencies of the admixture levels for all animals in Excel and plotted them in categories of 0.1 steps. We assigned 30 purebred Baoulé (global admixture levels ≥ 0.999 Baoulé) and 30 purebred Zebu (global admixture levels ≥ 0.987) as reference populations to investigate local admixture levels in 716 animals that were considered as potential crossbreds based on the sampling information. Animals found to be purebred (global admixture levels ≥ 0.995 Baoulé) were removed from the pool of crossbreds.

### Local Ancestry Estimation in Admixed Populations

Local Ancestry in Mixed Populations (LAMP) is a program for estimating locus-specific ancestries in admixed individuals, using allele frequencies of the reference populations (Sankararaman et al., 2008). We applied the LAMP-ANC mode implemented in LAMP and provided the estimated allele frequencies files for Baoulé and Zebu as the purebred ancestral populations. LAMP-ANC is a modification of the LAMP mode and shows higher accuracy allowing triple mixing to be estimated, while LAMP cannot determine frequencies for more than two ancestral populations (Sankararaman et al., 2008). The following parameters were set: admixture proportions (alpha) = 0.8 for Baoulé and ≥ 0.2 for Zebu based on the global ancestry estimation using ADMIXTURE program, number of generations since admixture (g) = 2 and recombination rate (r) = 10^–8^. We estimated the local ancestry proportion, as well as the “delta ancestry” with R in trypanosome positive and negative trypanosomosis animals following Khayatzadeh et al. (2016) using a custom script (see section “Data Availability”). The “delta ancestry” reflects the extreme fluctuations in ancestry differences across the genome, which are calculated by subtracting the genome-wide ancestry from locus-specific ancestry for each ancestry component. Such extreme fluctuations in ancestry differences are unlikely to have occurred by random genetic drift and potentially exhibit a selection signature in the admixed individuals (Tang et al., 2007). To identify significant deviations from the genome-wide average ancestry, we performed permutation tests (Doerge and Churchill, 1996) of the local ancestry proportions over the whole genome of admixed animals as proposed and carried out by Tang et al. (2007) in an admixed human population (Puerto Ricans) and replicated by Gautier and Naves (2011), Flori et al. (2014) in African Taurine, and Khayatzadeh et al. (2016) in composite cattle breed (Swiss Fleckvieh) to find significant thresholds for the deviations of local genetic ancestries from global ancestries. Separating animals with positive and negative trypanosomosis status, for each animal we concatenated the local ancestry estimations of all 29 autosomes and then permuted the circularized genome by cutting at a random location and rearranging the two resulting pieces of the genome for each individual independently. This type of permutation largely preserves the extent of Linkage Disequilibrium (LD), assuming that it is homogeneously distributed over the whole genome. We implemented 1,000 permutations. The distributions of maximum and minimum over all permutations were then used to define the 5 and 10% genome-wide thresholds levels that indicated significant deviation of the observed local ancestries from the genome-wide average ancestry (Tang et al., 2007; Gautier and Naves, 2011; Khayatzadeh et al., 2016).

### *F*_*ST*_ Outlier Analysis

We applied BayeScan 2.1 (Foll and Gaggiotti, 2008) to identify *F*_ST_ outlier loci putatively under selection between the trypanosome positive (*n* = 244) and negative (*n* = 266) crossbred animals using a cut-off at *p* < 0.05 corrected for a false discovery rate [FDR; (Benjamini and Hochberg, 1995)]. BayeScan uses a Reversible Jump Markov Chain Monte Carlo (RJ-MCMC) algorithm to obtain posterior distributions, with 100,000 iterations and a Burn-in length of 50,000 iterations. The regions within ± 0.5 Mb of the most significant SNPs were searched for any potential associated genes based on the ARS UCD1.2 Bos Taurus Genome Assembly on the NCBI database.

### Identification of Ancestry Informative SNPs for Effective Hybrid Detection

We aimed to identify SNPs with the highest *F*_ST_ differentiation between the 30 pure Baoulé and 30 pure Zebu. We re-filtered the original dataset for MAF < 10%, individual and genotype missingness < 10%, respectively. The *F*_ST_ values were calculated in PLINK following Weir and Cockerham (1984). With these we were able to provide a set of top 200 *F*_ST_ markers, which were then used as a starting point to manually remove markers less than 5 Mb to each other—preference given to higher *F*_ST_ markers. Based on this, we selected the top 15, 25, 50, and 100 SNPs, and extracted these for the crossbred animals and repeated the global admixture analysis (*K* = 2). We used the *cor* function in R to calculate the Pearson correlation coefficient (Pearson’s *r*) for pairwise determining the linear association between admixture levels (ancestry proportion) estimated based on the different sets of ancestry informative SNPs [all SNPs (35,952 SNPs) versus the top 100, 50, 25, 15 SNPs]. Significance of the Pearson’s *r* was assessed with the *P*-value from the Pearson Correlation Coefficient Calculator (Social Science Statistics, 2021).

## Results

### Global Admixture of All Animals

**FIGURE 2:**
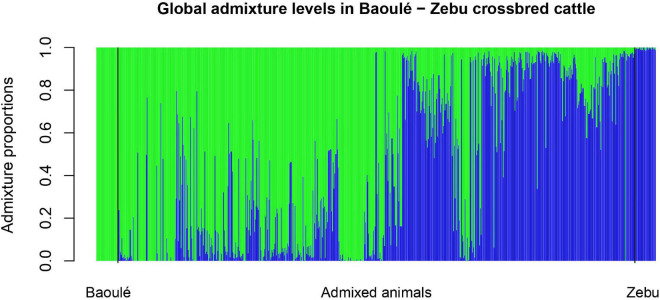
Global Admixture in Baoulé—Zebu crossbred cattle with the quality-filtered set of 28,034 single nucleotide polymorphisms (SNPs).

**FIGURE 3:**
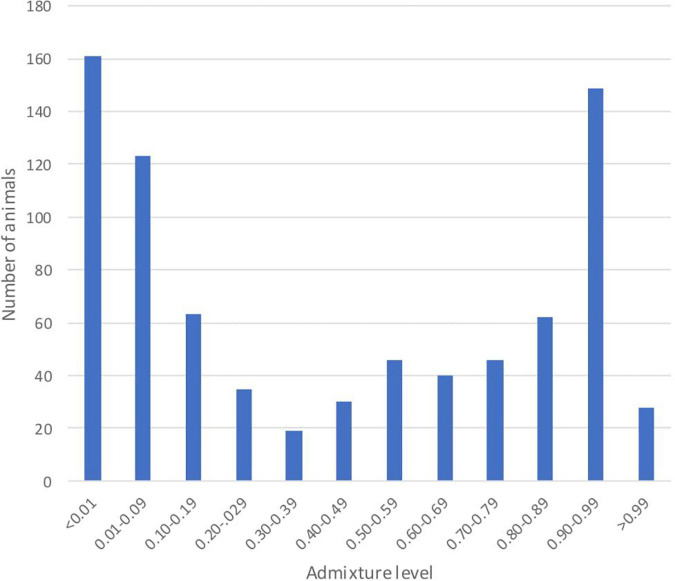
Distribution of the global admixture proportions for 802 animals with the full set of 31,612 single nucleotide polymorphisms (SNPs).

The individual admixture proportions using the full set of SNPs were estimated for all pure and admixed animals and are presented in Figure 2. The distribution of the global admixture proportions for the 802 animals is presented in Figure 3. Notably, we detected 91 cattle with a Baoulé ancestry > 0.995 among the presumed crossbreds, which we excluded from the subsequent analysis of local ancestry in admixed animals.

### Local Ancestry and the Delta Ancestry Across Chromosomes in Trypanosome Positive and Negative Crossbreds

**FIGURE 4:**
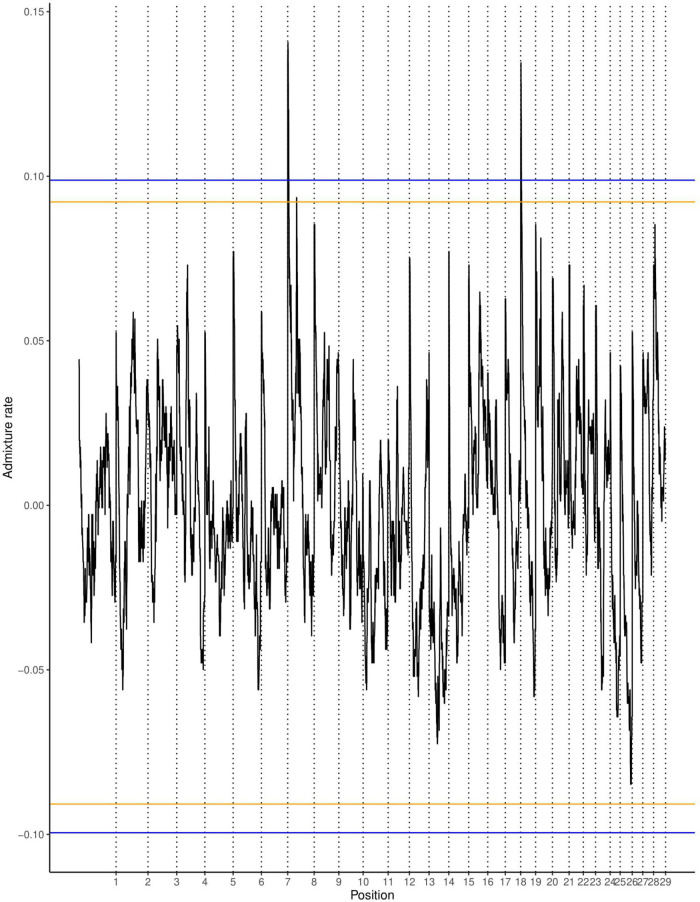
Local ancestry deviations based on the permutation threshold for the 244 positive crossbreds animals. Orange and blue lines signify the 5 and 10% genome-wide threshold.

**FIGURE 5:**
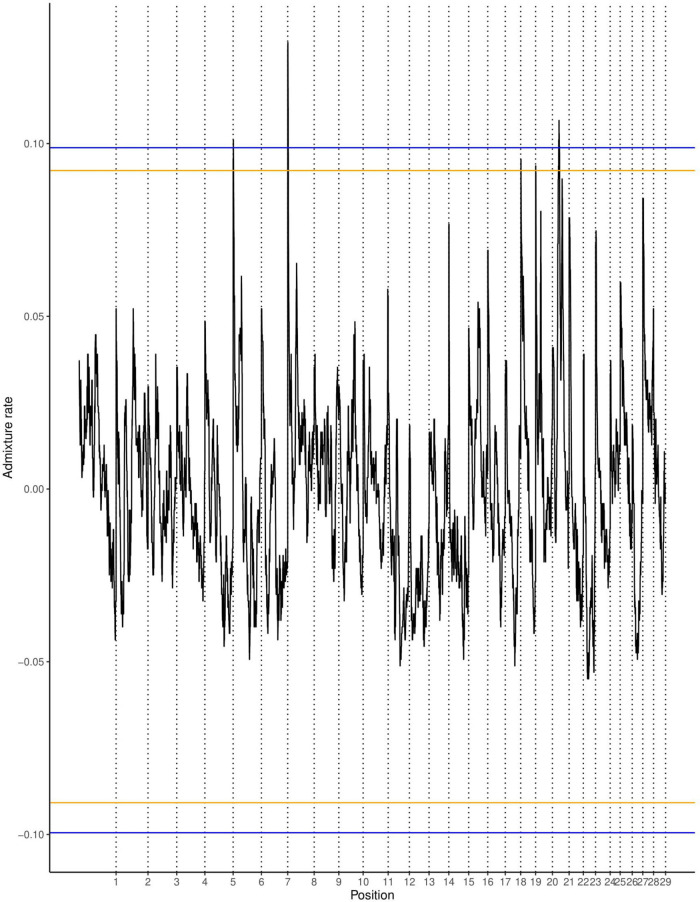
Local ancestry deviations based on the permutation threshold for the 266 negative crossbreds animals. Orange and blue lines signify the 5 and 10% genome-wide threshold.

**FIGURE 6:**
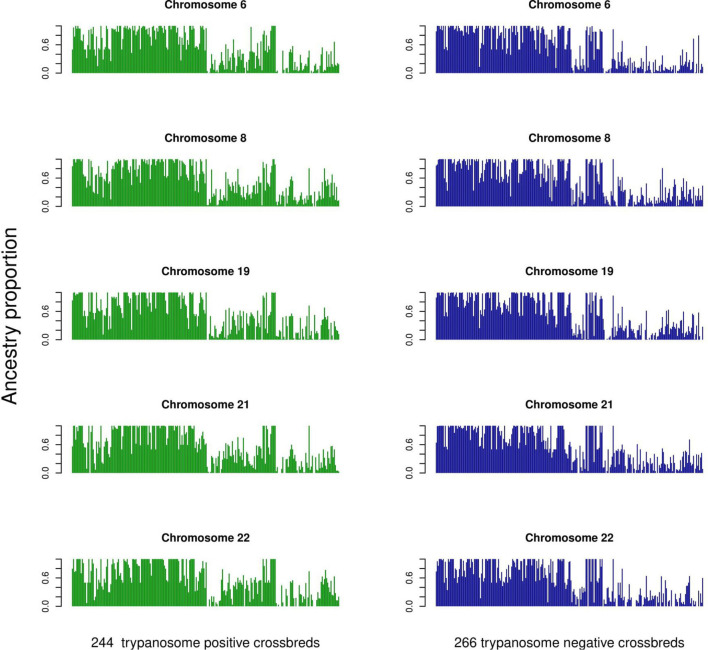
Individual admixture proportions across chromosomes 6, 8, 19, 21, and 22 for the 244 trypanosomose positive and 266 negative crossbreds as determined by LAMP.

**FIGURE 7:**
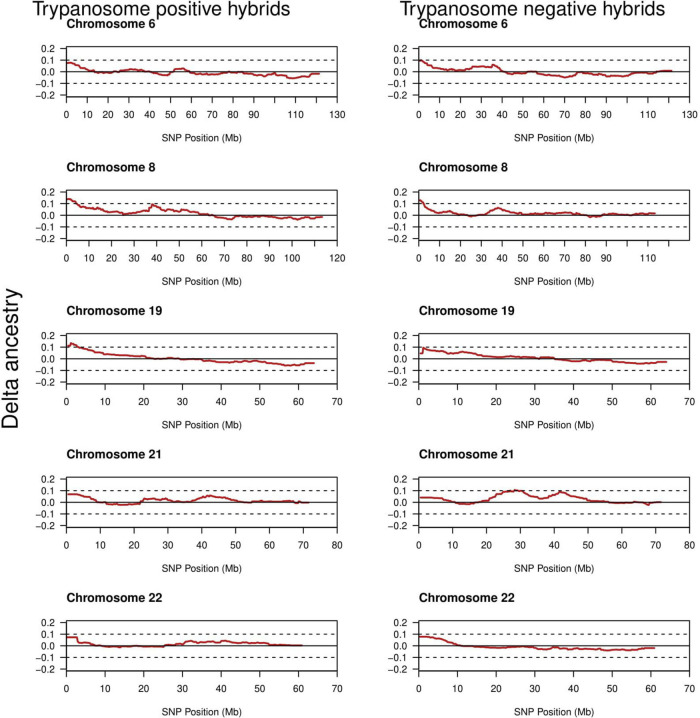
The delta ancestry across chromosomes 6, 8, 19, 21, and 22 for the 244 positive and 266 negative crossbreds trypanosomosis status. The red line shows the deviation.

The average ancestry estimation for every single SNP was performed across 29 autosomes for trypanosome positive and negative Baoulé x Zebu crossbreds, respectively. The permutation tests over all chromosomes indicated significant local ancestry deviation from the average (above the 5 and 10% genome-wide thresholds) in chromosomes 8 and 19 for trypanosome positive crossbreds (Figure 4), and in chromosomes 6, 19, 21, and 22 for trypanosome negative animals, respectively (Figure 5). The local admixture proportions for these chromosomes are presented in Figure 6 and for all other chromosomes in Supplementary Figures 1, 2. We further visualized the deviations from the average ancestry in the respective chromosomes and identified regions of higher delta ancestry (wide peaks) on chromosome 8 between 35–50 Mb and in chromosome 21 between 20–35 Mb and 40–50 Mb, respectively (Figure 7). These genomic regions might harbor candidate genes associated to tolerance or susceptibility of trypanosomosis.

### *F*_*ST*_ Outliers Between Trypanosome Positive and Negative Crossbreds

**FIGURE 8:**
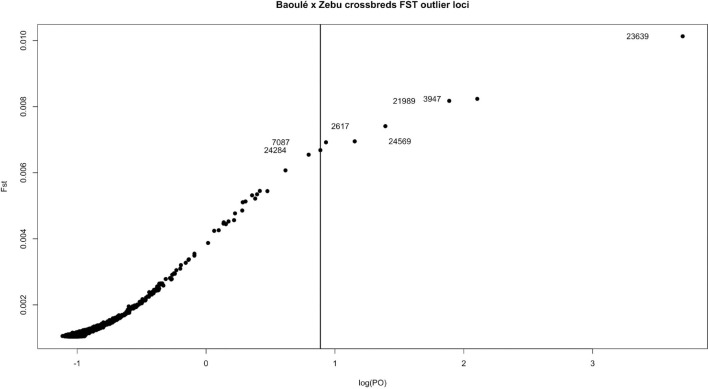
*F*_*ST*_ outliers between trypanosome positive and negative crossbreds. The vertical line shows the FDR corrected cut-off (*p* < 0.05); the outlier SNP names and positions are provided in Table 2.

We screened the genomes of the Baoule and Zebu crossbred animals for outlier SNPs with high *F*_ST_ values and disregarded the pure-bred Baulé and Zebu. Among these crossbred animals we grouped them in trypanosome positive and negative animals to avoid detection of breed differences unrelated to trypanosome tolerance. We detected seven variants with a FDR corrected threshold of *p* < 0.05 (Figure 8). The seven outlier SNPs with the highest levels of *F*_*ST*_ values were found in chromosomes 2, 3, 5, 20, 21, and 23, and are presented in Table 2, together with their neighboring genes. The positions of the SNPs were not located in regions with higher delta ancestry.

**TABLE 2:** The 7 outlier SNPs with the highest *F*_*ST*_ values.

**SNP**	**CHR**	**SNP name**	**Position**	**Genes**
2617	2	BovineHD0200021582	75210246	LOC100138101, LOC101902632
3947	3	BovineHD0300017052	56576857	HS2ST1, LMO4, ENSBTAG00000054817, and ENSBTAG00000052091
7087	5	ARS-BFGL-NGS-110363	108172899	CACNA1C, DCP1B, CACNA2D4, LRTM2, ADIPOR2, LOC101903199, ERC1, WNT5B, WNK1, RAD52, and FBXL14
21989	20	BovineHD2000008166	27620224	ISL1
23639	21	ARS-BFGL-NGS-22971	11067328	LOC107131341, NR2F2, and LOC101907985
24284	23	BovineHD4100016034	20992806	CD2AP, ADGRF2, ADGRF4, OPN5, and PTCHD4
24569	23	BovineHD2300012802	44142970	PHACTR1, HIVEP1, ADTRP, and EDN1

### Identification of the Most Ancestry Informative Markers

To reliably detect hybrids even with a small set of SNPs applicable for routine genetic monitoring, we selected the most ancestry informative markers resulting in the highest differentiation between Baoulé and Zebu cattle. The 100 SNPs with the highest divergence presented *F*_ST_ values ranging between 0.98 and 0.79 (Supplementary Table 1). We estimated admixture levels of the crossbred individuals using the top 15, 25, 50, and 100 SNPs (Supplementary Figure 5). The Pearson correlation coefficients *r* between the levels of admixture obtained with the full dataset of 35,952 SNPs and each of the sets of top SNPs were generally high and ranged between 0.949 (allSNPs/top15) and 0.990 (allSNPs/top100) (Table 3). All Pearson correlation coefficients were statistically significant (*p* < 0.001).

**TABLE 3:** Pearson’s correlation coefficient matrix displaying *r*^2^ values between the levels of admixture using the most ancestry informative markers compared to the full data set of 35,952 SNPs (allSNPs).

	**Top100**	**Top50**	**Top25**	**Top15**
allSNPs	0.990	0.984	0.970	0.949
Top100		0.994	0.980	0.962
Top50			0.986	0.966
Top25				0.984

## Discussion

### Global Admixture in the South-Western Taurine Cattle Population of Burkina Faso

The high amounts of global admixture observed in the taurine cattle population in the three studied departments of Burkina Faso indicated mixed genetic backgrounds of the cattle in Bouroum-Bouroum, Kampti and Loropeni (Figures 2, 3). The observed admixture levels within the departments are likely due to unrestricted mating among cattle of different genetic backgrounds. Long-distance migrations within and across countries, utilization of communal pastures, exchange of breeding animals, and uncontrolled mating facilitate constant gene flow. Houessou et al. (2019) explained this situation by lack of selection and high levels of gene flow due to cyclical cross-border cattle herd movements known as “transhumance” and extensive commercial transactions of cattle in the West African region.

The uncontrolled mating in extensive production systems, which are typically practiced in West Africa, can lead to the introgression of Zebu genes in the small taurine cattle population, which represents a threat to their genetic integrity (Dossa and Vanvanhossou, 2016), and might lead to a potential dilution of their trypanotolerance (Traoré et al., 2015; Albert et al., 2019). The increasing importance of Zebu in the South-western region of Burkina Faso might endanger Baoulé cattle in the long term. As the North, which is the preferred area for Zebu cattle, is hit by drought, increasing numbers of Zebu cattle breeders looking for pasture are moving to the South-west where the climate is quite favorable and grass is still abundant. Thus, suitable management is required for the sustainable use of local breeds, and recently community-based breeding programs (CBBP) for Baoulé cattle and their crossbreds have been implemented (Ouédraogo et al., 2020). Within the CBBP, Zoma et al. (2020) identified four distinct types of cattle production systems sedentary Lobi farms, sedentary crossbreed farms, semi-transhumant Fulani Zebu farms, and transhumant Fulani Zebu farms. The admixture between Zebus and Baoulé cattle observed in this study could be due to differences in the production systems. Furthermore, notable size differences between purebred and crossbred Baoulé were confirmed (Yougbaré et al., 2020) and breeders prefer to have large animals like Zebu cattle. As shown in Figure 2, we identified several purebred Baoulé cattle that had been considered as admixed based on the sampling information. These animals originated from the populations of Loropeni and Bouroum-Bouroum where the farmers have a preference for breeding purebred Baoulé (Zoma et al., 2020).

### Different Local Ancestry in Trypanosome Positive and Negative Baoulé x Zebu Crosses

In a recently admixed population, ancestral populations have been mixing for a relatively small number of generations, resulting in a new population with different proportions of their genome derived from the original parental populations (Khayatzadeh et al., 2016). Local ancestry analysis of admixed populations has been successfully used to detect recent selection in admixed Swiss Fleckvieh cattle (Khayatzadeh et al., 2016), as well as selection for Zebu introgressed regions in Colombian creole taurine cattle (Pitt et al., 2018). In our study, we applied this approach to identify significantly different local admixture levels and detected five chromosomes with higher deviation from the average ancestries, with an excess of Baoulé ancestry, which might account for a higher tolerance to trypanosomiasis. Similarly, Decker et al. (2014) investigated the population structure of domesticated cattle and calculated Asian indicine (*B. t. indicus*), Eurasian taurine, and African taurine (both *B. t. taurus*) ancestry proportions.

We applied an approach of significance testing and performed a permutation test of circularizing the genome by concatenating the SNPs of all autosomes in a single string, cutting this string once and rearranging the two resulting segments, as proposed by Tang et al. (2007). The permutation approach removes not only the effects of selection, but also the local effects of genetic drift; the threshold is considered non-conservative. Nevertheless, based on simulations (Tang et al., 2007) outliers are unlikely to be due to genetic drift. Therefore, this procedure is considered robust to find significant signals for selection while accounting for confounding effects of demographic history of the admixed cattle.

We found regions deviating from the average ancestry with a higher amount of Baoulé proportions on chromosomes 6, 8, and 19 in trypanosome negative individuals. A previous study (Noyes et al., 2011) identified *VAV1, PIK3R5, RAC1, VAV2, GAB2*, and *INPP5D* genes in chromosome 8 to be genes under selection in Muturu and N’Dama cattle breeds in response to trypanosomes infection. Surprisingly, we also found higher Baoulé ancestry in chromosome 8 (35–50 Mb) also in trypanosome positive cattle, which could indicate that these regions harbor beneficial Baoulé haplotypes, which are not connected to trypanosomosis tolerance. These regions might harbor genes of general importance for adaptation to the environment. Some canadidate genes on chromosome 6 at 71373513-71421283 (*PDGFRA*) and chromosome 19 at 41185975-41196948 (*CDC6*) for trypanotolerance in West African taurines have been found on these chromosomes (Tijjani, 2019) overlapping with the regions identified in our study. Furthermore, Smetko et al. (2015) identified chromosomes 7 and 22 as regions with the highest Baoulé ancestry proportion, similar to our results.

### Genes Under Potential Selection Identified by *F*_*ST*_ Outlier Tests

Identifying recent positive selection signatures in domesticated animals can provide information on beneficial mutations and their underlying biological pathways for economically important traits. Global *F*_*ST*_ values are one useful method to detect selection signatures across breeds (Biswas and Akey, 2006). The seven outlier SNPs, which we identified between trypanosome positive and negative crossbreds, were on chromosome 2, 3, 5, 20, 21, and 23. The chromosomes BTA 2, 3, 5, and 23 have previously been identified harboring common candidate genes in Muturu and N’Dama breeds linked to trypanotolerance in West African taurine population as well as selected candidate genes in Muturu cattle only (Tijjani et al., 2019). Functional annotation and enrichment analyses based on Reactome pathways in PANTHER ver 13.1 (Thomas et al., 2003) confirmed their relevance in response to trypanosome infection pathways. In our study, we identified other genes (Table 2) such as *LOC100138101, LMO4, LTRM2, ISL1, PTCHD4, and HIVEP1* as genes potentially responsible for trypanotolerance.

From previous studies genes such as *TICAM1, ARHGAP15, SLC40A1, GFM1*, and *INHBA* have been proposed as candidate genes for trypanotolerance on chromosomes 2, 3, and 5 (Dayo et al., 2009; Noyes et al., 2011). Bahbahani et al. (2018) identified the genes *LTA4H*, *IL7*, *IL15*, *FCN*, *LTA4H*, and *NFAM1* as potential targets of natural selection related to immunity in Sheko cattle, which are a mixture of Asian zebu and African taurine ancestry and considered a trypanotolerant breed with high potential for milk production.

### Ancestry Informative Markers to Detect Admixture for Routine Genetic Monitoring

The indigenous cattle breeds are disappearing because of indiscriminate crossbreeding by individual farmers, and schemes for genetic improvement developed without concern for preserving locally adapted breeds (Belemsaga et al., 2005). Many breeding programs or genetic improvement strategies in developing countries failed due to the lack of involvement of farmers in the different steps of implementation (Wurzinger et al., 2011). In many developing countries, livestock crossbreeding has been implemented with poor or no pedigree recording. Thus, ancestry informative markers would provide a great opportunity to estimate the level of admixture in a cost-effective way. Sölkner et al. (2010) proposed that individual admixture levels were estimated more accurately based on the genomic data using panels of pure reference animals, compared to estimation based on pedigree. (Getachew et al., 2017) indicated that the Ovine 50KSNP array is a powerful tool to identify small sets of AIMs for admixture studies in crossbred sheep populations in Ethiopia.

The minimum set of the 25 highest differentiating SNPs (Supplementary Table 1) can be used to develop an efficient competitive allele-specific PCR (KASP^TM^, LGC Group, United States) genotyping assay. Such an easy and fast genotyping array can be implemented at any laboratory equipped with Real-Time PCR machine and can be used for routine monitoring of hybridization in Baoulé cattle. Getachew et al. (2017) identified a total of 74 SNPs from the Ovine 50K SNP data as AIMs. The SNPs were selected based on their *F*_ST_ values showing the highest levels of allele frequency differentiation between the two parental breeds similar to our methodology. These AIMs provided close estimation with pedigree information. Correlation coefficient between breed level based on admixture estimates from 25 SNP data obtained in this study (*r* = 0.99; Table 3) was higher compared to the correlation value of 0.96 obtained from ∼500 AIMs suggested to predict breed composition in cattle (Frkonja et al., 2012) or the correlation values in the range of 0.89 to 0.96 reported for different human populations in prediction of admixture levels (Halder et al., 2008). Other studies (Judge et al., 2017) recommended at least 300 informative SNPs identified based on similar diversity parameters to be used for genomic-based breed composition prediction. However, as the purpose of our AIM set was to differentiate between only two ancestral breeds (Baoulé and Zebu) the number of 25 highest differentiating SNPs was sufficient to detect admixed individuals (Table 3 and Supplementary Figure 5). The existence of such a 25 SNP set allows their genotyping locally in Burkina Faso, providing a sustainable and low-cost solution to monitor admixture rates in these populations. We will further validate the 25 most AIMs in a larger group of confirmed crossbreds in Burkina Faso. Understanding the relationship between genetic admixture and performances is crucial for the success for local cattle breed conservation and crossbreeding programs. Ideally, a combination of pedigree and genomic information is used in breeding programs. Applying small sets of AIMs is a cost-effective option to estimate the levels of admixture in situations where pedigree recording is difficult like in Burkina Faso.

## Conclusion

In this study, we identified local ancestry proportions in genomic regions potentially related to trypanotolerance in front of a global admixture background. Based on a 10% genome-wide threshold exploring extreme deviations from the average distribution of delta ancestry, the chromosomes 6, 8, 19, 21, and 22 contained higher ancestral proportions of Baoulé cattle. Furthermore, we identified genes such as *LOC100138101*, *LMO4*, *LTRM2*, *ISL1*, *PTCHD4*, and *HIVEP1* as genes potentially responsible for trypanotolerance. Identification of genomic regions harboring genes related to trypanotolerance is a strong argument for conservation not only of Baoulé cattle, but all trypanotolerant breeds. The subsequent integration of these regions to genomes of non-trypanotolerant breeds *via* admixture provides a sustainable and effective use of these breeds, despite their lower production characteristics. As such, our study contributes to a better understanding of the genetic mechanism underlying trypanotolerance and will allow building a suitable breeding strategy for Baoulé cattle and their crossbreds in the south-western region of Burkina Faso.

The results indicate that the Bovine 50KSNP array is a powerful tool to identify small sets of AIMs as a cost-effective option to estimate the levels of admixture in situations where pedigree recording is difficult like in Burkina Faso. The minimum set of the 25 highest differentiating SNPs can be used to develop an efficient competitive allele-specific PCR assay.

## Data Availability Statement

Quality controlled Bovine 50k SNP chip data, including 31,612 SNPs of the 802 animals included in this study, were uploaded to DRYAD. The dataset has been assigned a unique identifier (doi: 10.5061/dryad.547d7wm7f).

## Ethics Statement

Ethical review and approval was not required for the animal study because Samples were collected during the official health monitoring activities of the APPEAR Project “Characterization and Sustainable Utilization of Local Cattle Breeds in Burkina Faso” approved by the Ministry of Agriculture and Irrigation Development (Ministère de l’Agriculture et des Aménagements Hydro-agricoles), Burkina Faso.

## Author Contributions

JS conceived the original idea of the study and together with GM, PB, BY, and NK further developed the idea and decided on the set of analysis. BY and PB did the statistical analysis and wrote the text. BY, DO, BZ, AS, SO-K, SM, HT, and ATr collected the data for the analysis and together with JS, GM, PB, NK, ATa, PO-W, MW, ATr, and OM critically reviewed the text. All authors approved the final version of the manuscript.

## Conflict of Interest

NK was employed by company SUISAG, Switzerland. The remaining authors declare that the research was conducted in the absence of any commercial or financial relationships that could be construed as a potential conflict of interest.

## Publisher’s Note

All claims expressed in this article are solely those of the authors and do not necessarily represent those of their affiliated organizations, or those of the publisher, the editors and the reviewers. Any product that may be evaluated in this article, or claim that may be made by its manufacturer, is not guaranteed or endorsed by the publisher.

## Supplementary Material

Supplementary Figure 1 The local ancestry estimation plot for 29 autosomes chromosomes for 266 trypanosome negative, in Baoulé X Zebu crossbred cattle, excluding CHR 6, 8, 19, 21, and 22 which are presented in Figure 6.

Supplementary Figure 2 The local ancestry estimation plot for 29 autosomes chromosomes for 244 trypanosome positive, in Baoulé X Zebu crossbred cattle, excluding CHR 6, 8, 19, 21, and 22 which are presented in Figure 6.

Supplementary Figure 3 The delta ancestry for 29 autosomes chromosomes for 244 trypanosome positive, in Baoulé X Zebu crossbred cattle excluding CHR 6, 8, 19, 21, and 22, which are presented in Figure 7.

Supplementary Figure 4 The delta ancestry for 29 autosomes for 266 trypanosome negative Baoulé X Zebu crossbred cattle excluding CHR 6, 8, 19, 21, and 22 which are presented in Figure 7.

Supplementary Figure 5 Admixture graphs of the top 100, 50, 25, and 15 ancestry informative SNPs.

Supplementary Table 1 The 100 highest differentiating SNPs according the *F*_*ST*_ value.
