# Supplementary material for: Local Ancestry to Identify Selection in Response to Trypanosome Infection in Baoulé x Zebu Crossbred Cattle in Burkina Faso
Source: Front Genet. 2021 Sep 27;12:670390. doi: 10.3389/fgene.2021.670390 (PMC8504455; doi:10.3389/fgene.2021.670390)
Supplement: Supplementary Figure 1 — The local ancestry estimation plot for 29 autosomes chromosomes for 266 trypanosome negative, in Baoulé X Zebu crossbred cattle, excluding CHR 6, 8, 19, 21, and 22 which are presented in Figure 6. [file Data_Sheet_1.PDF]

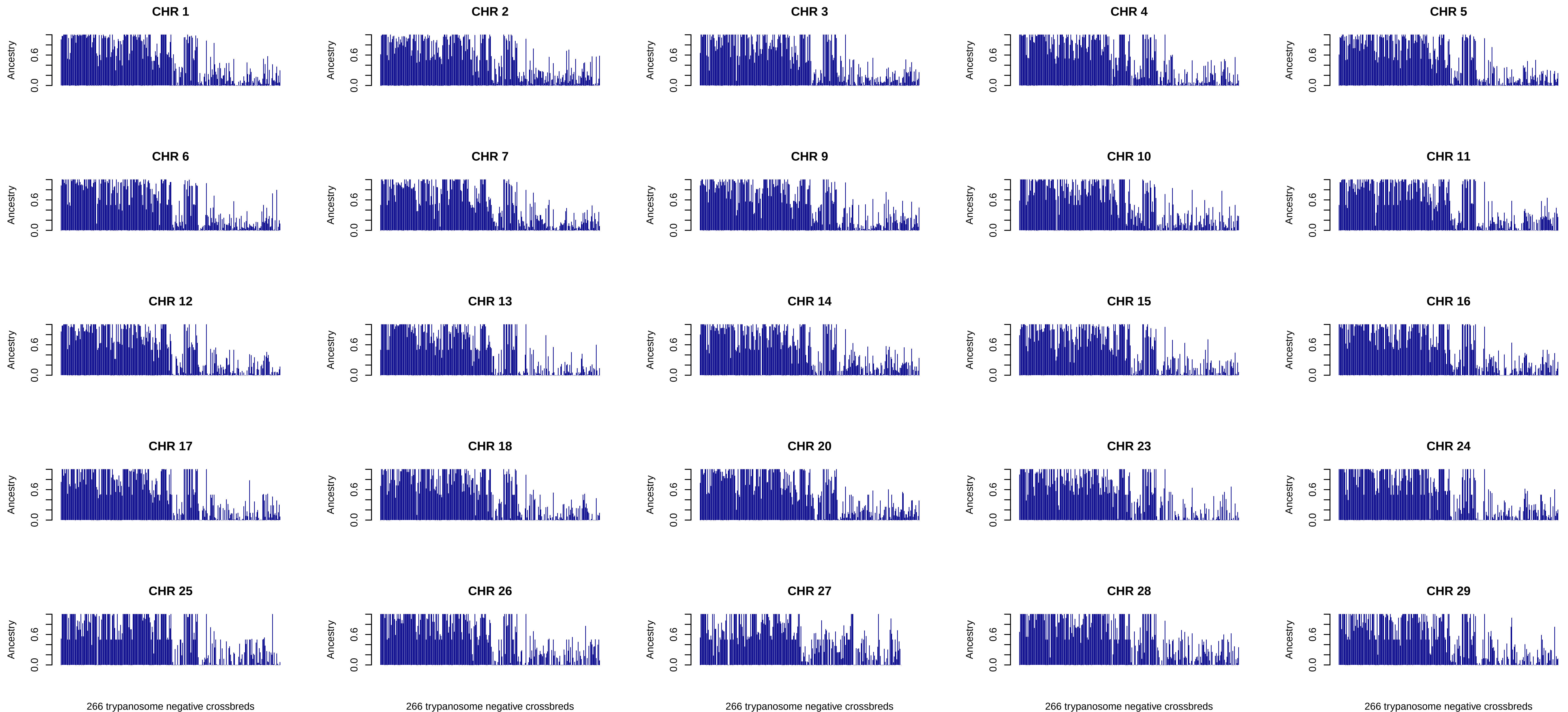
CHR 1
CHR 2
CHR 3
CHR 4
CHR 5
CHR 6
CHR 7
CHR 9
CHR 10
CHR 11
CHR 12
CHR 13
CHR 14
CHR 15
CHR 16
CHR 17
CHR 18
CHR 20
CHR 23
CHR 24
CHR 25
CHR 26
CHR 27
CHR 28
CHR 29
Ancestry
0.0
0.6
266 trypanosome negative crossbreds
