# Supplementary figures and images for: Local Ancestry to Identify Selection in Response to Trypanosome Infection in Baoulé x Zebu Crossbred Cattle in Burkina Faso
Source: Front Genet. 2021 Sep 27;12:670390. doi: 10.3389/fgene.2021.670390 (PMC8504455; doi:10.3389/fgene.2021.670390)

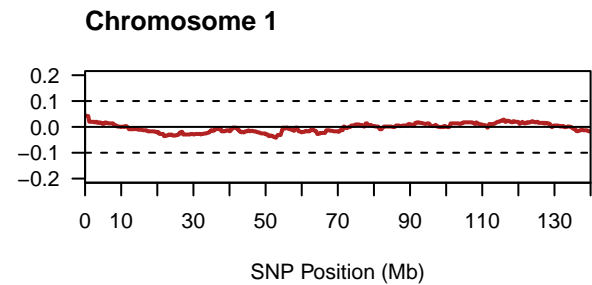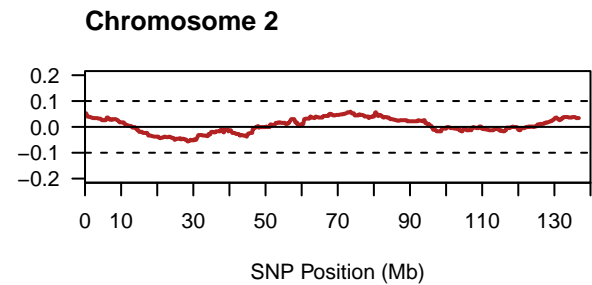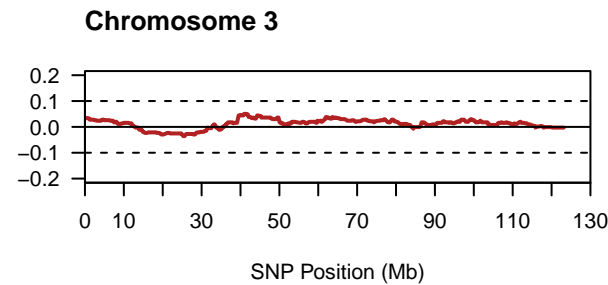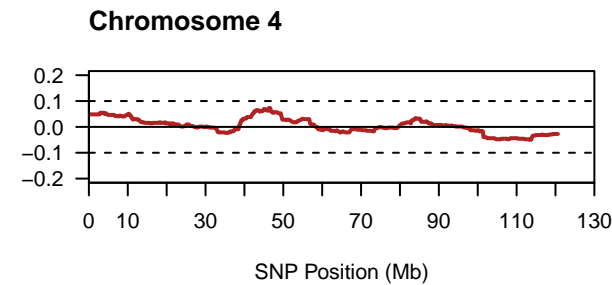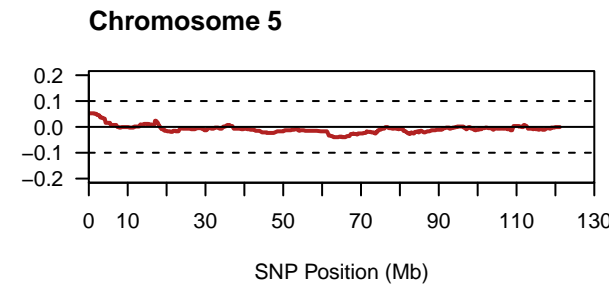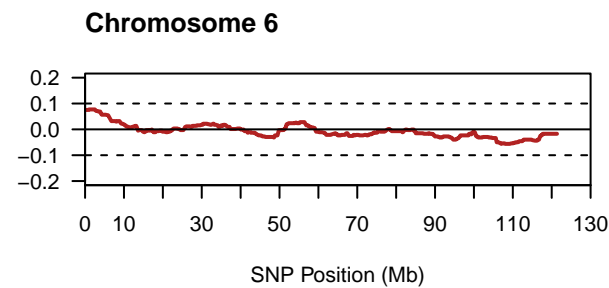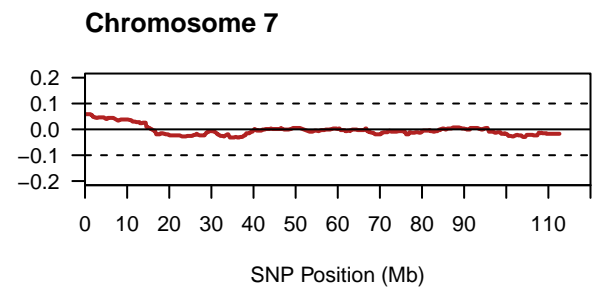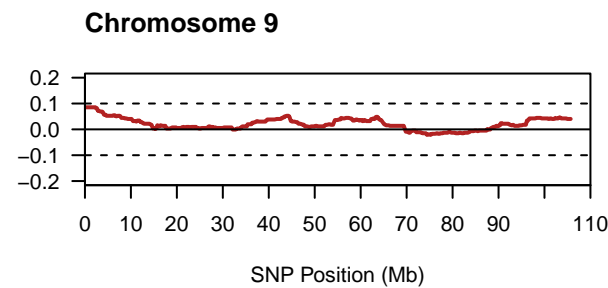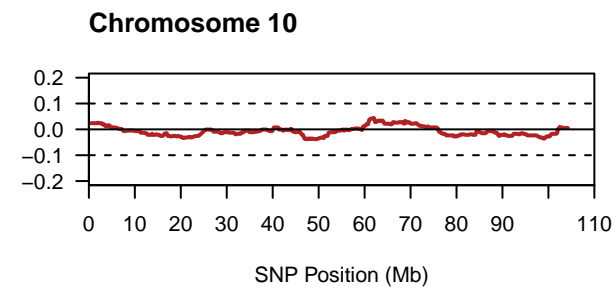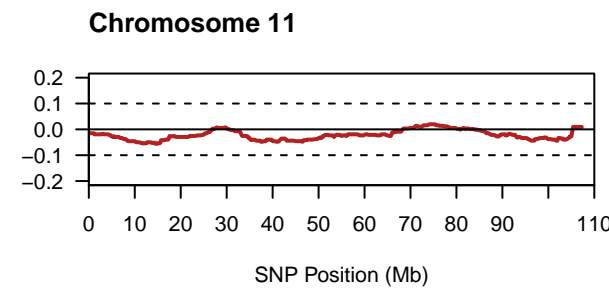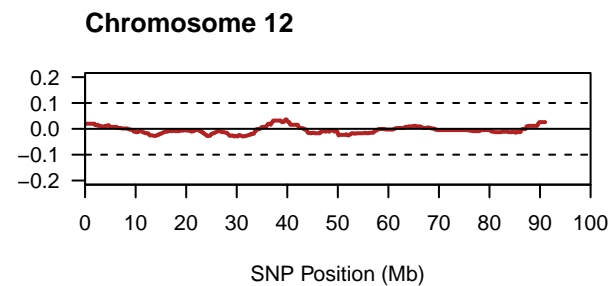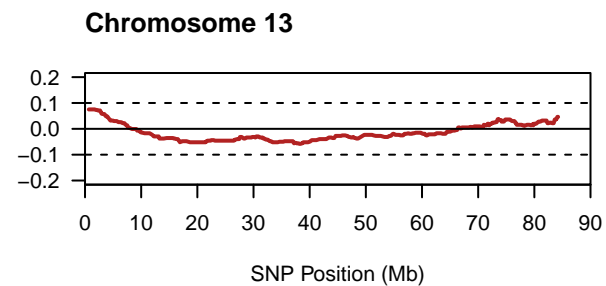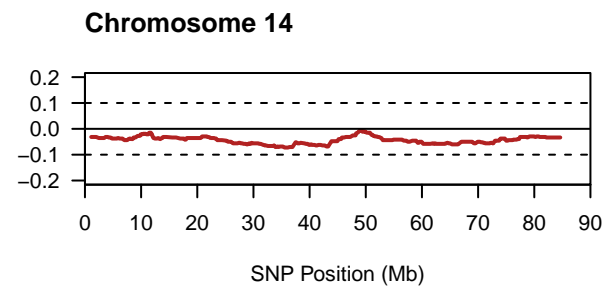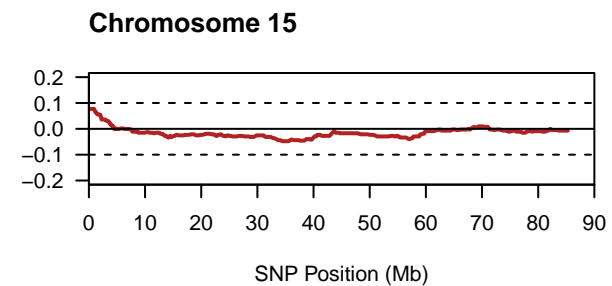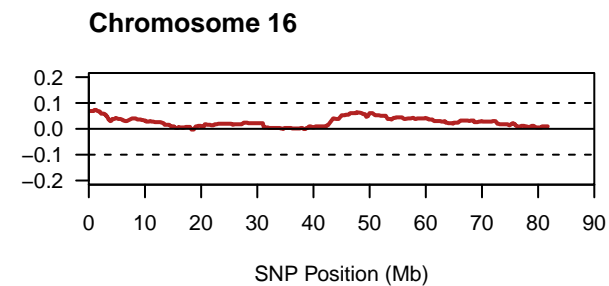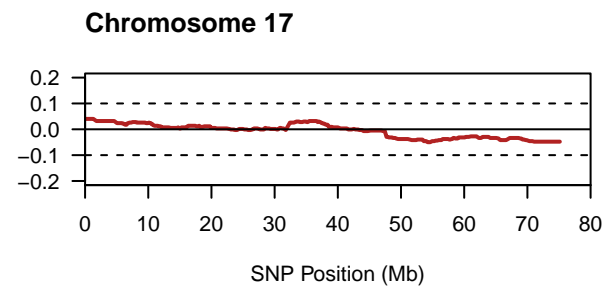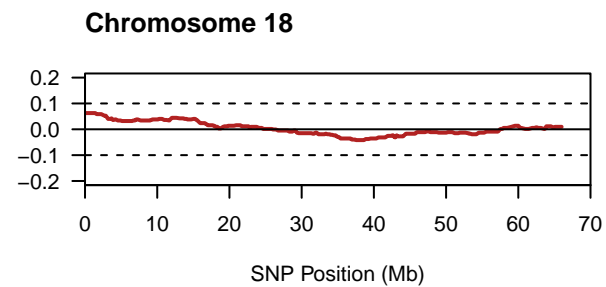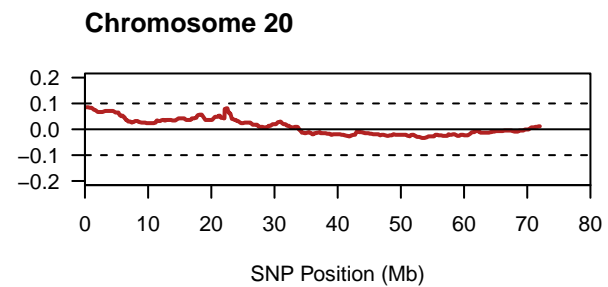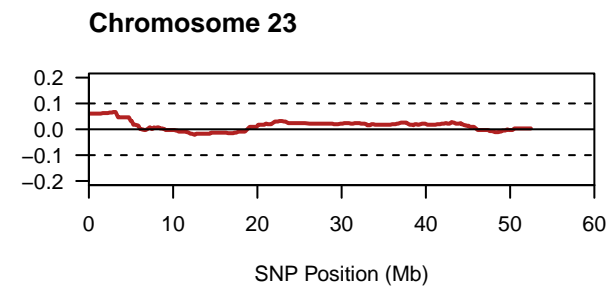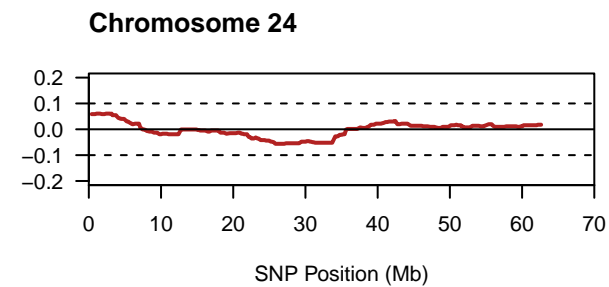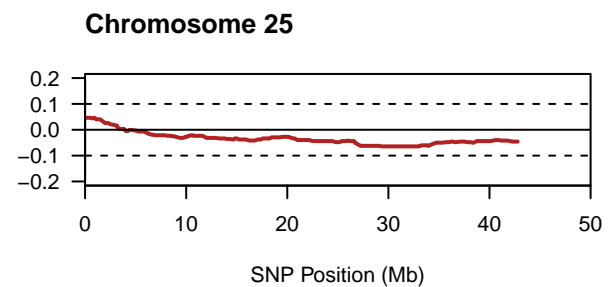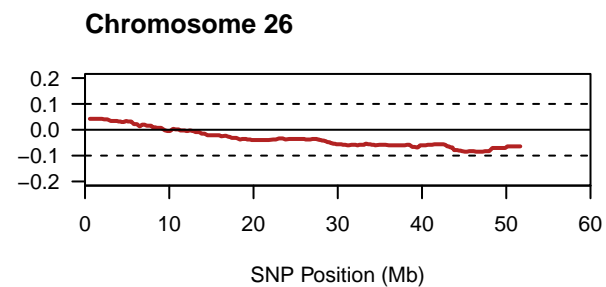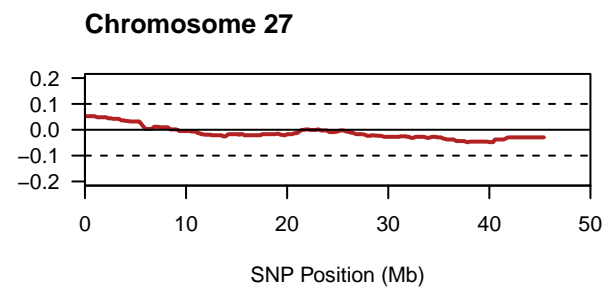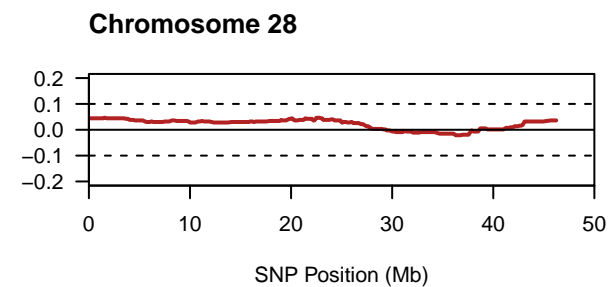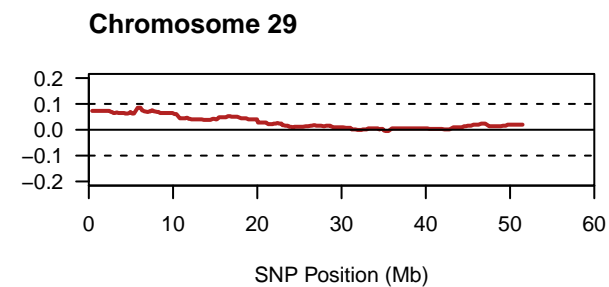

Supplement: Supplementary Figure 3 — The delta ancestry for 29 autosomes chromosomes for 244 trypanosome positive, in Baoulé X Zebu crossbred cattle excluding CHR 6, 8, 19, 21, and 22, which are presented in Figure 7. [file Data_Sheet_3.PDF]

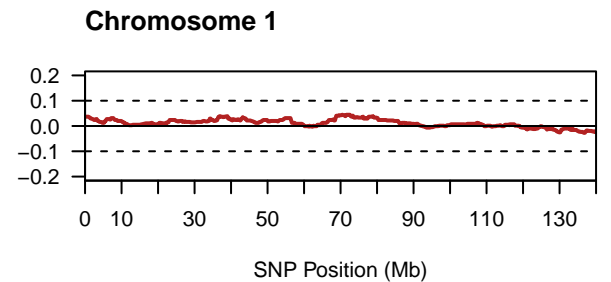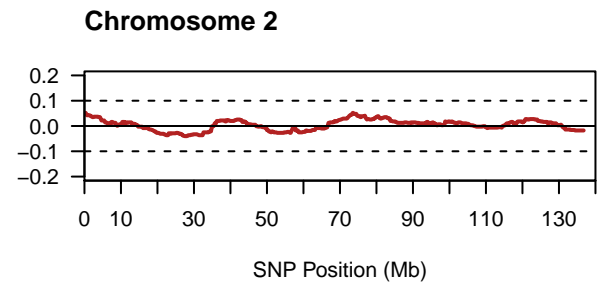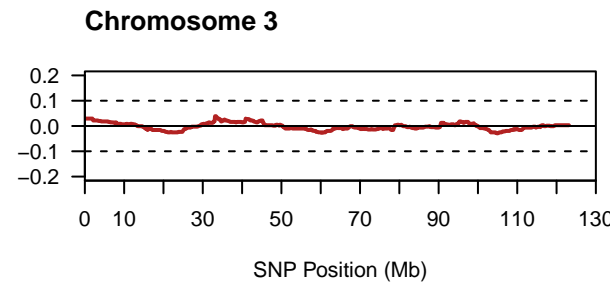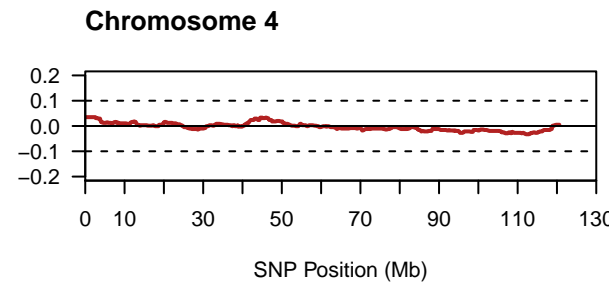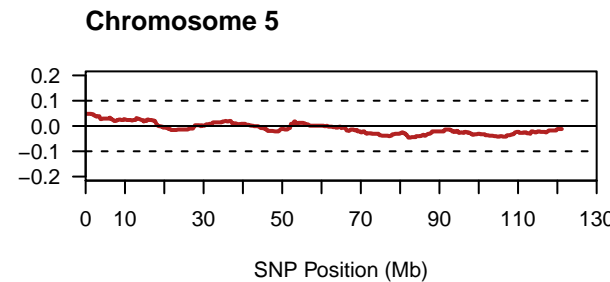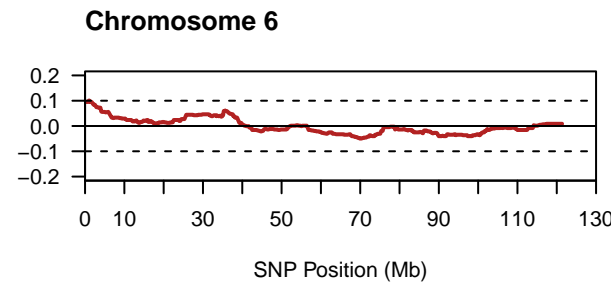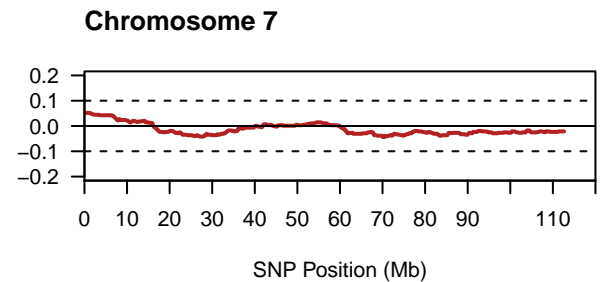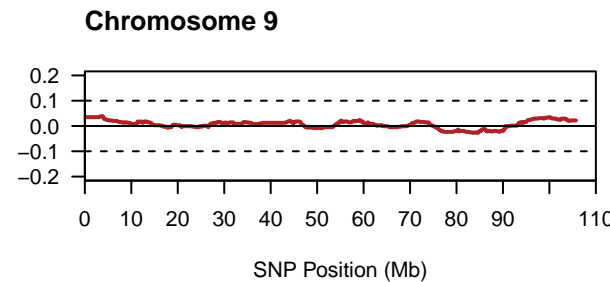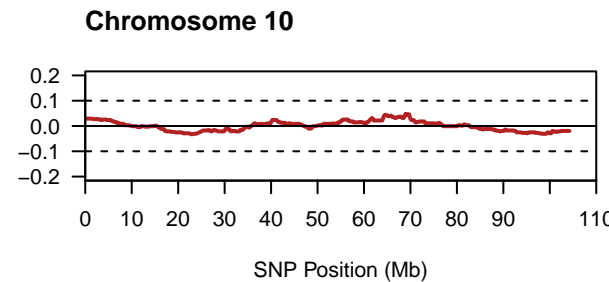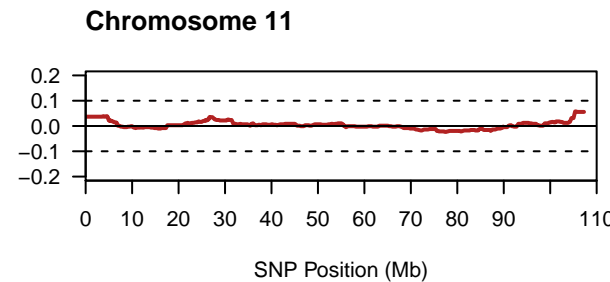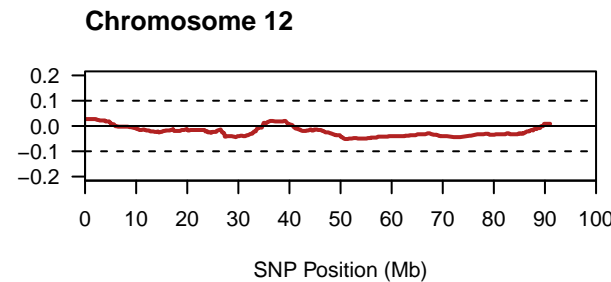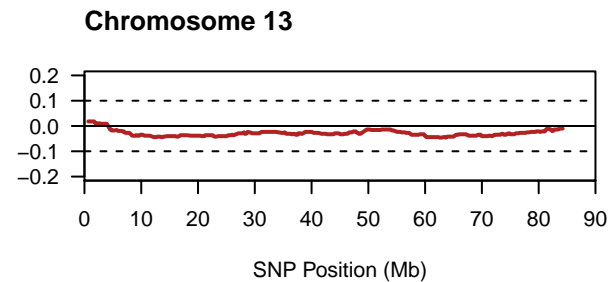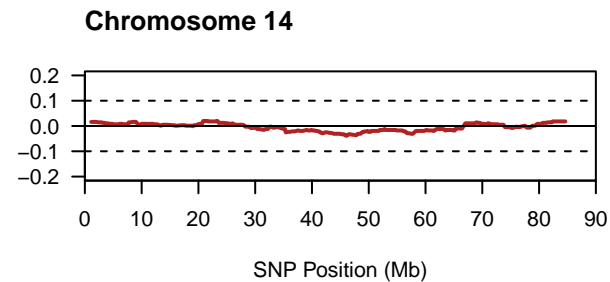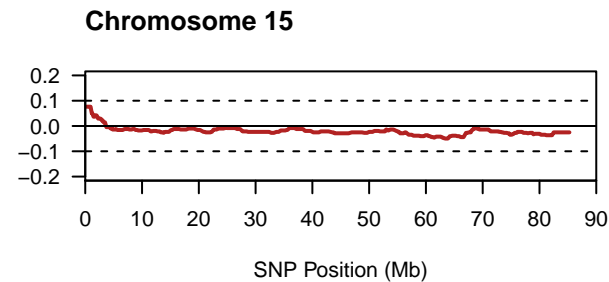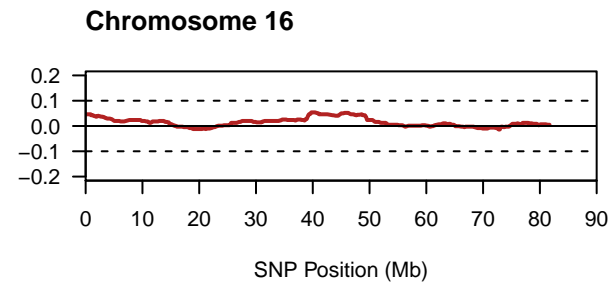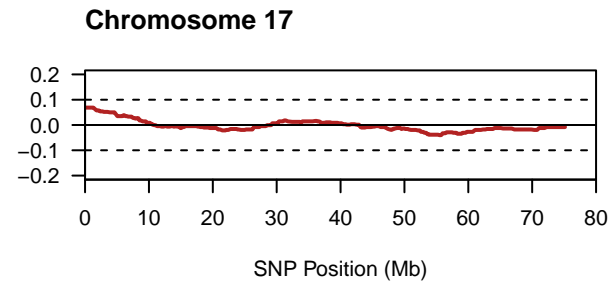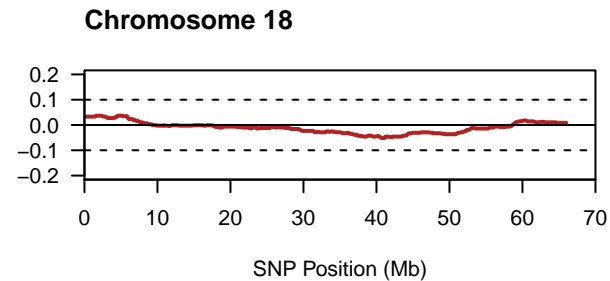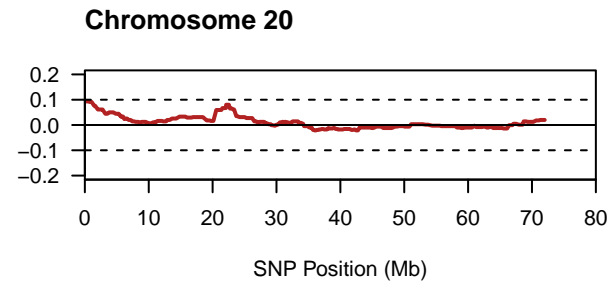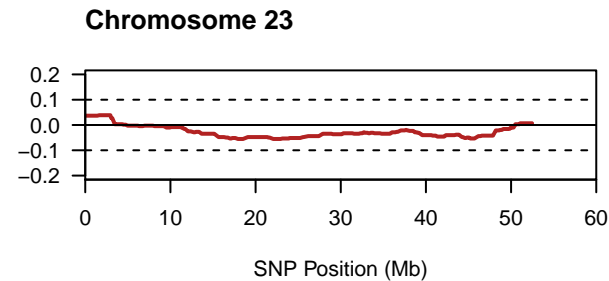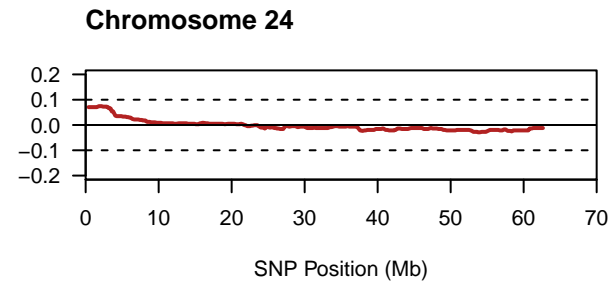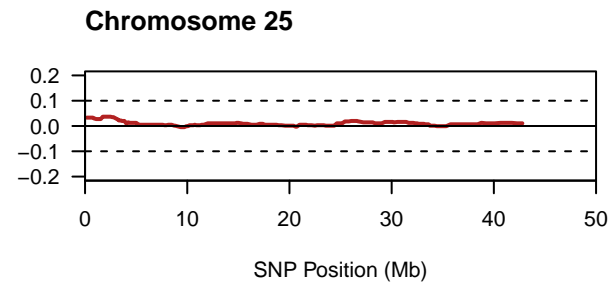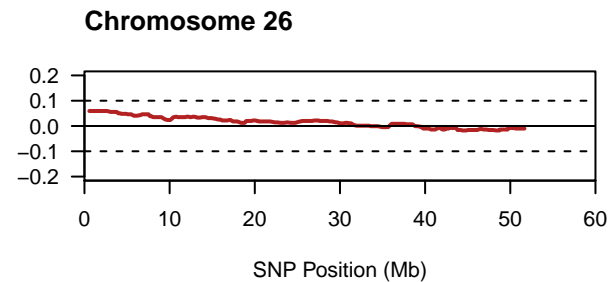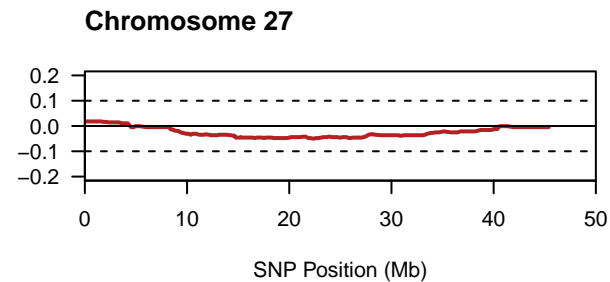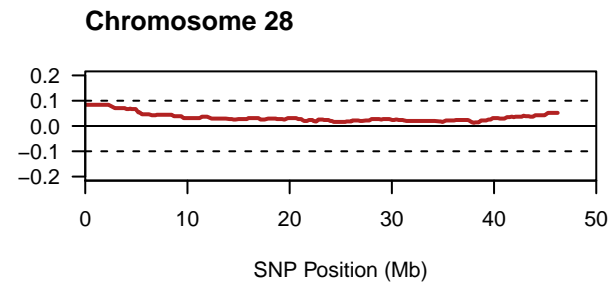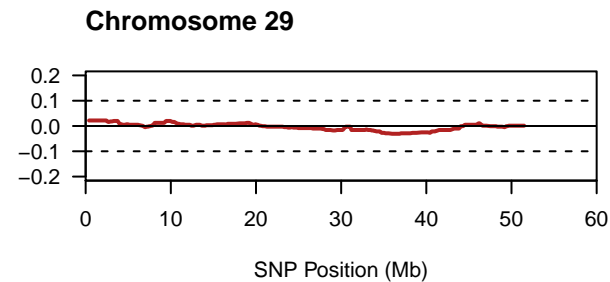

Supplement: Supplementary Figure 4 — The delta ancestry for 29 autosomes for 266 trypanosome negative Baoulé X Zebu crossbred cattle excluding CHR 6, 8, 19, 21, and 22 which are presented in Figure 7. [file Data_Sheet_4.PDF]
