# Supplementary material for: Local Ancestry to Identify Selection in Response to Trypanosome Infection in Baoulé x Zebu Crossbred Cattle in Burkina Faso
Source: Front Genet. 2021 Sep 27;12:670390. doi: 10.3389/fgene.2021.670390 (PMC8504455; doi:10.3389/fgene.2021.670390)
Supplement: Supplementary Figure 5 — Admixture graphs of the top 100, 50, 25, and 15 ancestry informative SNPs. [file Data_Sheet_5.PDF]

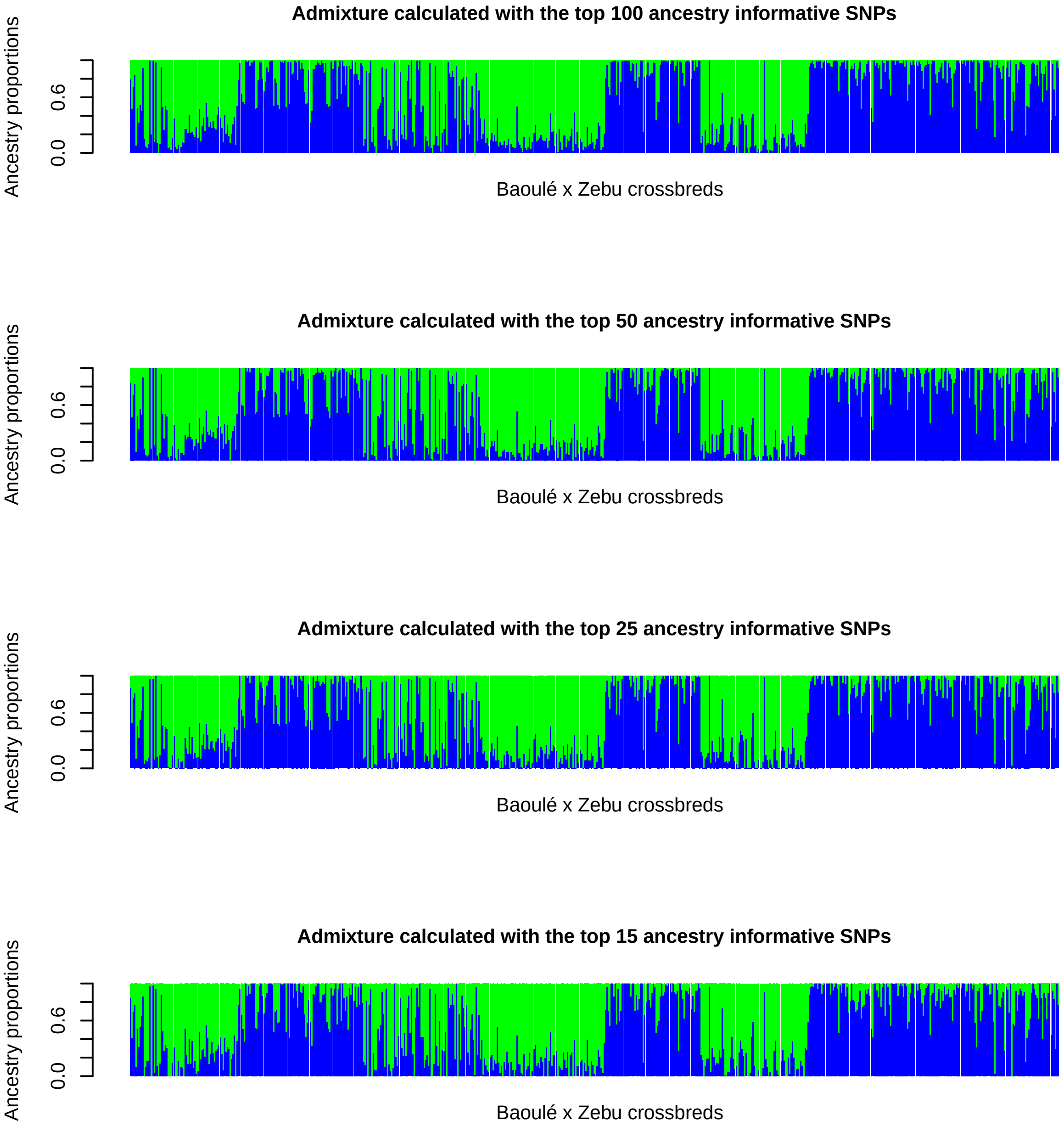
Admixture calculated with the top 100 ancestry informative SNPs
Ancestry proportions
0.0
0.6
Baoulé x Zebu crossbreds
Admixture calculated with the top 50 ancestry informative SNPs
Ancestry proportions
0.0
0.6
Baoulé x Zebu crossbreds
Admixture calculated with the top 25 ancestry informative SNPs
Ancestry proportions
0.0
0.6
Baoulé x Zebu crossbreds
Admixture calculated with the top 15 ancestry informative SNPs
Ancestry proportions
0.0
0.6
Baoulé x Zebu crossbreds
