# Supplementary material for: Local Ancestry to Identify Selection in Response to Trypanosome Infection in Baoulé x Zebu Crossbred Cattle in Burkina Faso
Source: Front Genet. 2021 Sep 27;12:670390. doi: 10.3389/fgene.2021.670390 (PMC8504455; doi:10.3389/fgene.2021.670390)
Supplement: Supplementary Table 1 — The 100 highest differentiating SNPs according the FST value. [file Table_1.DOCX]

**Table S1**: The 100 highest differentiating SNPs according the FST value

| **Chromosome number** | **SNP-Name** | **Position** | **FST-Value** |
| --- | --- | --- | --- |
| 5 | BovineHD0500014038 | 48684632 | 0.983051 |
| 5 | ARS-BFGL-NGS-4763 | 56585423 | 0.964319 |
| 7 | BovineHD0700014767 | 50596886 | 0.949183 |
| 13 | BovineHD1300012337 | 42418952 | 0.949183 |
| 3 | ARS-BFGL-NGS-67960 | 34263481 | 0.92997 |
| 13 | BovineHD1300010688 | 36956520 | 0.915361 |
| 13 | BovineHD1300014423 | 49869007 | 0.913063 |
| 18 | ARS-BFGL-NGS-11936 | 25653308 | 0.912438 |
| 5 | BovineHD0500018676 | 66809794 | 0.911919 |
| 7 | BovineHD0700019952 | 68127848 | 0.911919 |
| 12 | BovineHD1200008699 | 29542838 | 0.911919 |
| 7 | BovineHD0700015011 | 51739128 | 0.911294 |
| 5 | ARS-BFGL-NGS-100547 | 66784879 | 0.906898 |
| 2 | BovineHD0200008300 | 28396626 | 0.898467 |
| 3 | BovineHD0300016644 | 55064457 | 0.898467 |
| 4 | BovineHD0400011124 | 40104459 | 0.898467 |
| 12 | BTA-31783-no-rs | 19982250 | 0.898467 |
| 9 | BovineHD0900021248 | 76270904 | 0.897829 |
| 5 | BovineHD0500006844 | 23543325 | 0.895597 |
| 5 | BovineHD0500026582 | 93664518 | 0.894536 |
| 5 | BovineHD0500035017 | 119886893 | 0.894536 |
| 15 | BovineHD1500020125 | 69707369 | 0.893883 |
| 18 | Hapmap52308-rs29009652 | 54470798 | 0.893871 |
| 16 | ARS-BFGL-NGS-101656 | 41992472 | 0.891385 |
| 7 | BovineHD0700021133 | 71697239 | 0.881588 |
| 9 | BovineHD0900021283 | 76385110 | 0.881588 |
| 2 | BovineHD0200020386 | 70972553 | 0.880937 |
| 6 | BovineHD0600032704 | 115296748 | 0.880937 |
| 5 | Hapmap41950-BTA-72999 | 26082666 | 0.878144 |
| 7 | ARS-BFGL-NGS-15459 | 23756162 | 0.877495 |
| 7 | BovineHD0700008786 | 31011979 | 0.876847 |
| 2 | Hapmap30596-BTA-161397 | 69939162 | 0.874726 |
| 16 | BovineHD1600011216 | 39118839 | 0.874726 |
| 11 | BovineHD1100014651 | 49934803 | 0.864721 |
| 2 | ARS-BFGL-NGS-83221 | 70719167 | 0.864058 |
| 13 | BovineHD1300022886 | 79109584 | 0.863395 |
| 2 | BovineHD0200020335 | 70770040 | 0.862019 |
| 10 | BovineHD1000017894 | 61946034 | 0.860705 |
| 4 | BovineHD0400019264 | 70100877 | 0.859211 |
| 5 | BovineHD4100003413 | 9211843 | 0.857857 |
| 12 | BovineHD1200008803 | 29864049 | 0.855593 |
| 6 | BovineHD0600013238 | 48243519 | 0.855501 |
| 3 | Hapmap57282-ss46526266 | 95924827 | 0.85154 |
| 13 | BovineHD1300008042 | 27686629 | 0.84787 |
| 19 | UA-IFASA-6210 | 59715027 | 0.84787 |
| 10 | BovineHD1000025823 | 90327707 | 0.847194 |
| 11 | BovineHD1100011251 | 37930714 | 0.847194 |
| 6 | BovineHD4100004930 | 46808748 | 0.846518 |
| 20 | BovineHD2000004622 | 15282042 | 0.844948 |
| 12 | BovineHD1200007107 | 23573797 | 0.843281 |
| 14 | BovineHD1400012544 | 44320278 | 0.843281 |
| 11 | BovineHD1100014397 | 48966315 | 0.83987 |
| 4 | BovineHD0400022404 | 81050716 | 0.8392 |
| 3 | BovineHD0300035702 | 117337293 | 0.836942 |
| 7 | BovineHD0700015218 | 52757805 | 0.836942 |
| 9 | BovineHD4100007585 | 76516167 | 0.831153 |
| 11 | ARS-BFGL-BAC-11783 | 74603943 | 0.831034 |
| 19 | BovineHD1900006203 | 21678830 | 0.831034 |
| 3 | Hapmap51282-BTA-67903 | 55187147 | 0.830345 |
| 4 | BTA-70284-no-rs | 41895490 | 0.830345 |
| 12 | BovineHD1200008483 | 28644619 | 0.830345 |
| 20 | BTB-01300413 | 15253542 | 0.830345 |
| 10 | BovineHD1000011295 | 36041911 | 0.829655 |
| 10 | ARS-BFGL-NGS-28776 | 17377774 | 0.8219 |
| 13 | BovineHD1300016722 | 58378178 | 0.8219 |
| 18 | ARS-BFGL-NGS-15837 | 62591127 | 0.8219 |
| 12 | BovineHD1200014463 | 52404316 | 0.820535 |
| 7 | BovineHD0700014460 | 49961510 | 0.816846 |
| 4 | BovineHD0400014383 | 52024984 | 0.816167 |
| 7 | Hapmap35191-BES11_Contig367_1030 | 55144387 | 0.815489 |
| 5 | BovineHD0500018581 | 66379267 | 0.814215 |
| 6 | Hapmap33430-BTC-037618 | 41588847 | 0.814215 |
| 17 | BovineHD1700000823 | 3479572 | 0.814215 |
| 13 | BovineHD1300022893 | 79143377 | 0.813875 |
| 2 | BovineHD0200002852 | 10166949 | 0.813512 |
| 4 | BovineHD0400022134 | 80034734 | 0.813512 |
| 13 | BovineHD1300009238 | 31608219 | 0.813512 |
| 3 | BovineHD0300027088 | 94006679 | 0.812808 |
| 8 | BovineHD0800021888 | 72806242 | 0.812808 |
| 18 | BovineHD1800016466 | 56446320 | 0.812104 |
| 3 | Hapmap53284-rs29015774 | 55327770 | 0.809708 |
| 3 | BovineHD0300028475 | 98991477 | 0.808481 |
| 6 | BTB-00272418 | 96493695 | 0.808481 |
| 11 | BovineHD1100004099 | 12441952 | 0.807782 |
| 6 | BovineHD0600015761 | 57608133 | 0.806384 |
| 4 | BovineHD0400011578 | 41938042 | 0.803254 |
| 13 | BovineHD1300016713 | 58347218 | 0.803245 |
| 1 | BovineHD0100013052 | 45719178 | 0.800582 |
| 6 | BovineHD0600032874 | 115853782 | 0.800582 |
| 7 | BovineHD0700010226 | 35704892 | 0.800582 |
| 9 | BovineHD0900017167 | 62432661 | 0.800582 |
| 11 | Hapmap40862-BTA-100125 | 62144546 | 0.800582 |
| 6 | BovineHD0600012731 | 46730204 | 0.798352 |
| 5 | BovineHD0500025003 | 88134041 | 0.797661 |
| 1 | BovineHD0100003512 | 11155914 | 0.797414 |
| 2 | BovineHD0200002816 | 9928973 | 0.797414 |
| 4 | BovineHD0400033226 | 114860030 | 0.797414 |
| 14 | BovineHD1400014933 | 52870232 | 0.797414 |
| 15 | BovineHD1500012190 | 43894485 | 0.797414 |
| 16 | BTB-01226007 | 32591482 | 0.797241 |
